# Effects of Mindfulness–Acceptance–Insight–Commitment (MAIC) Training on Stress and Sleep Quality in Elite Swimmers: A Randomized Controlled Mixed-Methods Trial

**DOI:** 10.3390/bs16071068

**Published:** 2026-06-29

**Authors:** Ning Su, Bingyan Zhang, Xiyu Zhou, Jiayu Hu, Wei Liang, Dong Wang

**Affiliations:** 1 School of Physical Education, Shenzhen University, Shenzhen 518060, China; 2500201003@mails.szu.edu.cn (B.Z.); 13691807926@163.com (X.Z.); 2400201011@mails.szu.edu.cn (J.H.); 2 Mindful Movement and Health Joint Lab, School of Physical Education, Shenzhen University, Shenzhen 518060, China

**Keywords:** mindfulness, MAIC, elite swimmers, psychological stress, sleep quality, mixed methods

## Abstract

Elite swimmers are exposed to sustained high training loads, early-morning sessions, and restricted recovery opportunities, all of which may increase psychological strain and compromise sleep. This study examined the effects of an 8-week Mindfulness–Acceptance–Insight–Commitment (MAIC) program, embedded within routine high-load training, on athlete-specific psychological stress, subjective sleep quality, and mindfulness in elite swimmers. A randomized controlled mixed-methods design was used. Thirty elite swimmers from a provincial high-performance program in China were randomly assigned to an MAIC group or a usual-practice control group (*n* = 15 per group). Quantitative outcomes were assessed at baseline, post-intervention, and three-month follow-up using the Athlete Psychological Strain Questionnaire, salivary cortisol, the Pittsburgh Sleep Quality Index, and the Athlete Mindfulness Questionnaire. Semi-structured interviews were conducted with all athletes in the MAIC group after the intervention. Mixed-design ANOVAs revealed significant Group × Time interactions for athlete-specific psychological stress, salivary cortisol, sleep quality, and mindfulness. Compared with the control group, the MAIC group showed lower psychological strain and cortisol, better subjective sleep quality, and higher mindfulness at post-intervention. At follow-up, improvements in psychological stress and mindfulness remained evident relative to baseline, whereas lower salivary cortisol and more favorable self-reported sleep quality remained evident relative to the control group. Qualitative findings further showed that MAIC was experienced as feasible, low-burden, and readily integrated into the training context. Athletes described attentional resets, acceptance-based responses to discomfort, and brief post-session or pre-sleep practices as helpful for regulating cognitive reactivity and arousal. Overall, MAIC appears to be a culturally grounded and practically viable adjunct strategy for supporting psychological regulation and self-reported sleep quality in elite swimmers during demanding training periods.

## 1. Introduction

Regular physical activity (PA) is a cornerstone of mental and physical well-being, with convergent epidemiological evidence linking greater PA to lower risk of common mental health problems. A large dose–response meta-analysis of prospective cohorts shows that even sub-guideline volumes of PA are associated with substantially reduced incident depression, underscoring PA as a population-level protective factor for psychological health (Pearce et al., 2022). Beyond mental health, multiple reviews indicate that exercise confers benefits for sleep, including improved overall sleep quality and reductions in sleep latency among adults, reinforcing the view that movement-based behaviors and restorative sleep are reciprocally supportive pillars of health (Banno et al., 2018).

Elite sport, however, represents a distinct exposure context in which high training loads, compressed schedules, and competition pressures can erode these expected benefits. The International Olympic Committee’s consensus statement highlights that mental health symptoms and disorders are common in elite athletes, impair performance, and require systematic, program-level responses that integrate identification, prevention, and management (Reardon et al., 2019). Within this context, sleep is particularly vulnerable. In swimming, a discipline characterized by early-morning and twice-a-day sessions, objective monitoring demonstrates that early-morning training substantially curtails sleep opportunity and actual sleep duration, raising concerns about cumulative sleep restriction during heavy training phases (Ashby et al., 2024; Sargent et al., 2014a, 2014b). Reviews in elite athletes further document that inadequate sleep is common and consequential, with plausible impacts spanning recovery, cognition, immunity, and submaximal performance, while fluctuations in training load can alter sleep parameters and contribute to non-functional overreaching when recovery is insufficient (Halson, 2014).

Within this milieu, low-resource psychological strategies that can be embedded within routine training may offer additive protection for mental health and sleep without altering the underlying physical training prescription. Mindfulness-based interventions (MBIs) are increasingly adopted in sport as context-adaptable approaches that cultivate nonjudgmental present-moment awareness, acceptance of internal experiences, and values-consistent action. Contemporary syntheses in athletic populations report improvements in mindfulness and stress-related outcomes, alongside promising effects on performance-relevant variables, suggesting that MBIs can help athletes manage cognitive–emotional load during high-demand periods (Y. Wang et al., 2023). Mechanistically, mindfulness may lower pre-sleep cognitive and somatic arousal, which are key drivers of sleep disturbance, thereby facilitating sleep onset and continuity. The experimental and clinical literature indicate that mindfulness training reduces pre-sleep arousal and improves subjective sleep quality, and athlete-focused studies show similar short-term effects following mindfulness inductions after intensive evening training (Birnkraut et al., 2025; Ong et al., 2012). The Mindfulness–Acceptance–Insight–Commitment (MAIC) approach belongs to the broader family of mindfulness- and acceptance-based sport interventions, and shares several core processes with the Mindfulness–Acceptance–Commitment (MAC) approach and ACT-informed sport interventions, including present-moment attention, acceptance of internal experiences, cognitive defusion or decentering, values clarification, and committed action (F. L. Gardner & Moore, 2004; F. Gardner & Moore, 2007; Su et al., 2019). However, MAIC differs from more traditional mindfulness-based interventions and Western-developed MAC protocols in two related ways. First, rather than delivering general mindfulness practices as a stand-alone stress-management program, MAIC is explicitly designed for athletes and is embedded into concrete training and competition scenarios. Second, MAIC extends MAC by adding an explicit Insight component derived from Chinese cultural and sport-psychology traditions. In MAIC, Insight is intended to help athletes loosen rigid cognitive sets, decenter from obsessive or perfectionistic performance evaluations, and flexibly reappraise the relationship between immediate training adversity, personal athletic values, and socially embedded sport commitments (Su et al., 2019, 2024). Thus, the theoretical novelty of MAIC lies not in replacing established mindfulness-acceptance mechanisms, but in culturally and sport-contextually elaborating them through insight-based value integration and training-embedded committed action. In principle, MAIC may be particularly suited to elite swimming because swimmers routinely train under repetitive, fatigue-laden, and highly structured conditions, including early-morning sessions, twice-a-day schedules, dense microcycles, and continual exposure to pace feedback and bodily discomfort. These conditions create frequent moments of attentional drift, evaluative rumination, and post-session arousal, but they also provide clear behavioral anchors for brief mindfulness-based practice, such as between-set resets, breath anchoring, stroke-cue attention, cooldown down-regulation, and pre-sleep routines. Within a high-PA environment, where training volume and intensity remain unchanged, an embedded MAIC program may therefore confer additional benefits beyond those attributable to PA alone: attenuating athlete-specific psychological stress while improving perceived sleep quality by dampening pre-sleep arousal and reinforcing recovery-protective behaviors. This additive, “exercise-plus-mindfulness” framework aligns with calls to integrate mental health strategies into high-performance programs without imposing major logistical burdens or compromising sport-specific preparations (Reardon et al., 2019).

Despite growing interest, rigorous randomized evidence for embedded mindfulness programs delivered during periods of high training load in elite swimmers remains limited, and few studies couple quantitative outcomes with qualitative inquiry into athlete experiences and pragmatic uptake. Addressing this gap requires designs that preserve ecological validity, maintaining the routine training plan, while testing whether mindfulness yields incremental gains in stress and sleep over and above usual practice. Randomized controlled trials complemented by embedded qualitative components are well positioned to capture both effect magnitudes and the contextual mechanisms that shape adherence, perceived utility, and transfer to daily training tasks (Halson, 2014; Walsh et al., 2021).

The present randomized controlled mixed-methods trial therefore evaluated the effects of an 8-week MAIC program delivered during high-load training in elite swimmers, with training prescriptions held constant across groups. Primary outcomes were athlete-specific psychological stress and sleep quality assessed at baseline (T0), post-intervention (T1), and three-month follow-up (T2). Secondary outcomes included mindfulness level, reflecting the intervention’s putative mechanism of action. In line with the literature linking PA to mental health and sleep, and with evidence that mindfulness reduces pre-sleep arousal and stress, the following hypotheses were specified: (i) relative to usual practice, MAIC would reduce athlete-specific psychological stress at post-intervention and maintain reductions at follow-up; (ii) MAIC would improve sleep quality on the same timeline; (iii) MAIC would increase mindfulness level.

Collectively, this study situates mindfulness within the realities of elite swimming with early starts, dense training microcycles, and sustained physical loading, rather than apart from them. By testing MAIC as an embedded, low-resource mental health strategy under high training load, the trial addresses a practical question for coaches, practitioners, and athletes: can a context-attuned mindfulness program deliver incremental gains for stress and sleep without modifying the physical activity dose that is central to performance preparation? The findings are intended to extend the evidence base for integrative, athlete-centered approaches that align psychological skills training with the demands and recovery constraints of high-performance sport (Bender & Lambing, 2024; Halson, 2014; Walsh et al., 2021).

## 2. Materials and Methods

### 2.1. Study Design

This study employed a two-arm, parallel-group randomized controlled trial embedded within a mixed-methods framework. The quantitative component evaluated an 8-week Mindfulness–Acceptance–Insight–Commitment (MAIC) program delivered during routine high-load training in elite swimmers, with assessments at baseline (T0), post-intervention (T1; week 8), and three-month follow-up (T2; week 20). Primary outcomes were athlete-specific psychological stress and sleep quality; mindfulness level served as a secondary outcome reflecting the intervention’s putative mechanism. To contextualize and extend the trial findings, semi-structured one-to-one interviews were conducted after T1 to capture athletes’ lived experiences, perceived barriers and enablers, and pragmatic uptake of MAIC in daily training. The physical activity (training) prescription (periodized pool and dry-land work) was not manipulated; both groups adhered to the pre-planned squad program to preserve ecological validity. The study was approved by the Medical Ethics Committee of Shenzhen University (Approval No. PN-202400106) and conducted in accordance with the Declaration of Helsinki. Data were de-identified prior to analysis, and confidentiality was assured.

### 2.2. Participants

Participants were 30 elite swimmers recruited from a provincial high-performance program in China and randomized 1:1 to MAIC group (*n* = 15; *M_age_* = 17.27, *SD_age_* = 3.58; females = 7, males = 8) or usual-practice control group (*n* = 15; *M_age_* = 15.27, *SD_age_* = 5.43; females = 5, males = 10). All athletes were in full squad training throughout the study and held National Class I or higher qualifications under the Chinese athlete technical grading system (i.e., Class I, Master, or International Master). These nationally recognized classifications are awarded on the basis of official competitive performance standards, indicating that participants were high-performance swimmers within China’s provincial-to-national elite pathway rather than recreational or sub-elite athletes. No participants discontinued the trial or missed outcome assessments (retention = 100%).

#### 2.2.1. Eligibility Criteria

Inclusion criteria were: (i) continuous team-based training during the study period; (ii) athletic qualification at National Class I or higher; (iii) no structured mindfulness/meditation in the previous 12 months; (iv) no current use of antidepressants or anxiolytic medication; and (v) medical clearance with no cardiopulmonary contraindications. Athletes (and parents/guardians for minors) provided written informed consent and agreed to complete all assessments. Recent major injury, diagnosed sleep disorders requiring medical treatment, or participation in concurrent psychological interventions targeting stress or sleep were grounds for exclusion.

#### 2.2.2. Sample Size

The sample size was determined within the practical constraints of recruiting from a single intact provincial high-performance swimming squad. An a priori power analysis using G*Power 3.1, informed by a high-quality RCT in competitive athletes (Cohen’s d = 0.87), indicated that ≥14 participants per group would achieve 70% power at *α* = 0.05. This power threshold is below the conventional 80–90% level commonly recommended for fully powered clinical or behavioral intervention trials. Accordingly, the 70% threshold was used as a feasibility-constrained planning estimate in the context of a fixed elite-athlete sampling frame, rather than as evidence that the trial was optimally powered for definitive efficacy testing. This approach is consistent with methodological guidance suggesting that sample size justification in early-stage or feasibility-oriented behavioral intervention research should be aligned with the study purpose, target population, intervention context, and implementation constraints, while avoiding definitive efficacy claims based on small samples (Beets et al., 2021).

In the present study, the eligible athlete pool was limited by roster size, training availability, and inclusion criteria, and the intervention was deliberately embedded within the team’s routine high-load training environment to preserve ecological validity. As a result, 30 eligible athletes were ultimately enrolled and were randomized equally to the MAIC group and the usual-practice control group (*n* = 15 per group). Retention was 100%, and all randomized athletes provided complete data at T1 and T2.

Accordingly, the present study should be interpreted as a small-sample, ecologically embedded RCT providing preliminary evidence on the feasibility and potential effects of MAIC in elite swimmers, rather than as a fully powered efficacy trial. The effect estimates are therefore reported and interpreted cautiously, alongside their convergence with qualitative data.

#### 2.2.3. Recruitment and Allocation

Recruitment proceeded via team briefings with coaching and medical staff. After eligibility screening and baseline assessment, a computer-generated random sequence allocated athletes 1:1 to groups, with allocation concealment ensured using sealed opaque envelopes prepared by personnel independent of data collection. Given the nature of the intervention, participants and facilitators were not blinded; outcome assessors and data analysts were blinded to group assignment. For the qualitative strand, all 15 athletes in the MAIC group volunteered and completed individual interviews within two weeks of T1.

### 2.3. Intervention and Control Condition

#### 2.3.1. Intervention

MAIC is a sport-specific, culturally adapted mindfulness-based intervention (MBI) developed in China for competitive athletes (Si et al., 2020, Su et al., 2019, 2024). The intervention comprised an 8-week MAIC curriculum embedded within routine high-load training. Weekly group sessions (≈90 min) were delivered, supplemented by brief micro-practices scheduled around training (e.g., between-set resets, post-session down-regulation) and daily home practice (≈15 min) recorded in logs. Core components included: (i) mindfulness/attention training (present-moment focus under load); (ii) acceptance and cognitive defusion (reducing experiential avoidance and reactivity); (iii) insight (loosening rigid cognitive sets and fostering flexible, values-aligned appraisal); and (iv) values-based committed action (transfer to training tasks and pre-sleep routines). In the present swimming-adapted MAIC curriculum, the Insight component was operationalized as practices designed to help athletes notice rigid interpretations of fatigue, off-pace performance, perfectionistic self-evaluation, or short-term training discomfort, and then reappraise these experiences in relation to longer-term athletic values and recovery-protective choices. In this sense, Insight was intended to bridge mindfulness and acceptance with committed action by helping athletes move from ruminative or evaluative reactions toward more flexible, values-aligned responses during training and recovery. For elite swimmers, these practices were linked to common training situations, such as early-morning sets, repeated high-intensity intervals, post-session rumination, and pre-sleep cognitive activation. Intervention fidelity was supported by a session manual. To monitor intervention adherence, session attendance was recorded by the intervention instructor at each MAIC session using attendance logs, and home-practice minutes were self-reported weekly using brief adherence checklists. The eight-week MAIC curriculum outline is provided in Table 1.

#### 2.3.2. Control

The control group continued the pre-planned periodized training program and received routine squad education on health and recovery provided by coaching/medical staff. This usual-practice education consisted of routine team-based reminders regarding training attendance, injury prevention, nutrition and hydration, cooldown, and general recovery arrangements. It did not include structured mindfulness training, formal psychological skills training, stress-management sessions, or a systematic sleep-hygiene program. No individualized psychological counseling or sleep-focused intervention was provided as part of the control condition. Control athletes were asked to avoid initiating new mind–body programs (e.g., mindfulness, yoga nidra, meditation apps) for the study duration. To mitigate inequity and minimize contamination after the trial, control athletes were offered access to the MAIC materials/sessions after T2.

#### 2.3.3. Physical-Activity Context

The trial did not manipulate training load. Both groups maintained the pre-planned periodized program (pool and dry-land), verified by attendance records and coaching logs. The interpretation of effects is therefore situated within a stable high-load training environment.

### 2.4. Measures

#### 2.4.1. Athlete-Specific Psychological Stress

Athlete-Specific Psychological Stress was assessed with the Athlete Psychological Strain Questionnaire (APSQ) (Rice et al., 2020). A validated Chinese version was used (Tan et al., 2021). The APSQ comprises 10 items across three dimensions: (i) self-regulation difficulties; (ii) performance concerns; and (iii) externalized coping. Items are rated on a 5-point Likert scale (1 = “never” to 5 = “always”), higher composite scores indicate greater athlete-specific strain. In the present study, internal consistency Cronbach’s *α* = 0.72/0.75/0.74 at T0/T1/T2.

As a physiological correlate of stress and hypothalamic–pituitary–adrenal (HPA) axis activity, salivary cortisol was collected immediately upon awakening (within 5 min of wake time, prior to getting out of bed, food/drink, caffeine, tooth-brushing, or exercise) at each assessment wave (T0/T1/T2) (Hellhammer et al., 2009; Stalder et al., 2016). Because only one saliva sample was collected immediately after awakening on a single sampling day at each assessment wave, the cortisol measure represents single-day, single-sample awakening salivary cortisol concentration. Athletes recorded exact waking time; sampling was scheduled on routine training days (non-competition) and repeated on the same weekday within ±7 days across waves to minimize diurnal/weekly variance. Compliance with the awakening collection protocol was checked using athlete-reported waking-time and sampling records, and all retained samples were reported to have been collected within the required 5 min post-awakening window. Athletes were also asked to report acute illness, fever, medication use, or atypical training exposure around the sampling period. However, menstrual cycle phase was not systematically controlled. Samples were refrigerated promptly, then frozen at −20 °C and batch-assayed in duplicate using a commercially available Human Cortisol ELISA kit validated for saliva samples (BY-JZF0159; Nanjing BYabscience Technology Co., Ltd., Nanjing, China). According to the manufacturer’s instructions, the lower limit of detection was <1.0 nmol/L. Salivary cortisol concentrations were expressed in nmol/L. Intra- and inter-assay coefficients of variation were maintained within manufacturer limits (≤15%).

#### 2.4.2. Sleep Quality

Sleep quality was assessed with the Pittsburgh Sleep Quality Index (PSQI) (Buysse et al., 1989). A validated Chinese version was used (Tsai et al., 2005). The PSQI yields seven component scores summed to a global score (0–21), with higher global scores indicating poorer sleep quality. In the present study, PSQI scores were used to index perceived sleep quality rather than objectively measured sleep parameters, such as actigraphy- or polysomnography-derived sleep duration, sleep efficiency, or sleep architecture. In the present study, internal consistency Cronbach’s *α* = 0.74/0.83/0.83 at T0/T1/T2.

#### 2.4.3. Mindfulness

Mindfulness was assessed with the Athlete Mindfulness Questionnaire (AMQ) (Zhang et al., 2017). The AMQ comprises 16 items across three dimensions: (i) present-moment attention; (ii) awareness; and (iii) acceptance. Items are rated on a 5-point Likert scale (1 = “never true” to 5 = “always true”); higher composite scores indicate greater mindfulness. In the present study, internal consistency Cronbach’s *α* = 0.71/0.72/0.77 at T0/T1/T2.

### 2.5. Interview Guide

Each semi-structured interview was guided by a structured topic guide (Table 2), with follow-up probes used as needed. All interviews were conducted within 1–2 weeks after T1, one-to-one (≈30–45 min) in a quiet, private room, and were audio-recorded with consent.

### 2.6. Data Analysis

#### 2.6.1. Quantitative Data Analysis

All quantitative analyses were conducted using SPSS 27.0 (IBM Corp., Armonk, NY, USA). Statistical significance was set at *p* < 0.05. Prior to inferential analyses, data were screened for missing values, outliers, and distributional assumptions. Normality was formally assessed using Shapiro–Wilk tests and visual inspection of Q-Q plots. To examine the effects of the intervention over time, 2 (Group) × 3 (Time) mixed-design ANOVA (i.e., two-way repeated measures analyses of variance) were performed for each outcome variable, with Group (MAIC vs. control) as the between-subjects factor and Time (T0, T1, T2) as the within-subjects factor. The main effects of Group and Time, as well as the Group × Time interaction, were tested. Mauchly’s test was used to examine the sphericity assumption. When sphericity was violated, Greenhouse–Geisser correction was applied. Where significant interaction effects were observed, Bonferroni-adjusted post hoc comparisons were conducted to examine within-group changes across time and between-group differences at each assessment point. Effect sizes are reported as partial eta squared (*η*^2^). Given the small sample size, exploratory sensitivity analyses were conducted by including age as a covariate to examine whether the pattern of Group × Time effects was robust to age adjustment. Because the study was not powered for covariate-adjusted models, these analyses were interpreted cautiously and used only as sensitivity checks. In addition, partial *η*^2^ values were interpreted cautiously because effect-size estimates from small samples may be unstable and may overestimate the magnitude of the true population effect. Accordingly, interpretation emphasized the direction and pattern of effects, confidence intervals for between-group differences, and convergence with qualitative findings, rather than relying solely on statistical significance or effect-size magnitude.

#### 2.6.2. Qualitative Data Analysis

Qualitative data were analyzed using reflexive thematic analysis within a critical-realist, interpretive stance to explain how athletes experienced and enacted MAIC under high training load (Braun & Clarke, 2006, 2019). All audio-recorded interviews were verbatim transcribed, and transcripts were anonymized (P01–P15). A hybrid deductive–inductive coding strategy was applied: deductive sensitizing concepts drew on the topic guide and MAIC logic, while inductive coding captured unanticipated content. Coding and theme development were led by the primary analyst through an iterative and reflexive process, moving back and forth between data extracts, the developing codes, and the full dataset. Initial codes were first generated at the semantic and latent levels, then organized into candidate subthemes and higher-order themes. A coding tree was developed to document the progression from initial codes to subthemes and final themes. A second analyst acted as a critical discussion partner through regular analytic conversations and peer debriefing to challenge interpretations, refine conceptual clarity, and strengthen the credibility of theme development. Codes were iteratively clustered into themes, which were reviewed against extracts and the full corpus, then defined and named, with attention to discordant or less favorable cases. An audit trail was maintained, including coding notes, reflexive memos, code-to-theme development records, and summaries of analytic discussions. NVivo supported data management and coding. The primary analyst had training in sport psychology and familiarity with mindfulness-based sport interventions, which supported contextual interpretation but also created potential assumptions about the value of MAIC. To address this, reflexive memos and peer-debriefing discussions were used to examine how prior theoretical commitments and expectations may have shaped coding and theme development. Rigor was further enhanced through brief member checks at interview close, and judgment of sample adequacy by information power given the focused aim and specific sample (Malterud et al., 2016; Lincoln & Guba, 1985). Consistent with reflexive thematic analysis, emphasis was placed on thoughtful interpretation, transparency of analytic decisions, and researcher reflexivity rather than on coding consensus alone.

#### 2.6.3. Mixed-Methods Integration

Quantitative and qualitative strands were first analyzed separately and then integrated at the interpretation and reporting stages. Integration followed a weaving and joint-display approach, in which quantitative findings for psychological stress, salivary cortisol, sleep quality, and mindfulness were compared with qualitative themes describing athletes’ experiences of MAIC. The purpose of integration was explanatory: qualitative findings were used to help interpret how and why quantitative changes may have occurred in the training context, rather than to serve as independent confirmation of intervention efficacy.

A joint display was developed to compare quantitative findings, related qualitative themes, integrated inferences, and discordant or limiting cases. During integration, convergence, complementarity, and areas of divergence between strands were examined. Particular attention was given to less favorable or limiting cases, such as participants who described insight-related content as initially abstract, difficulty practicing after double training sessions, constrained sleep opportunity before very early training, or the need for booster sessions and more stroke-specific cues. Because qualitative interviews were conducted only with MAIC participants, the integrated interpretation primarily explains intervention experiences within the MAIC group and does not allow a balanced between-group qualitative comparison of intervention and control-group perspectives.

## 3. Results

### 3.1. Quantitative Results

Preliminary analyses indicated no missing data. Shapiro–Wilk tests and visual inspection of Q-Q plots did not indicate severe departures from normality for the quantitative outcomes. No significant between-group differences were observed at baseline in demographic or study variables (all *p* > 0.05). However, given the small sample size, baseline comparability was interpreted cautiously rather than as evidence of full equivalence. The standardized baseline difference (standardized mean difference, SMD) for age was 0.43, indicating a small-to-moderate age imbalance, with the MAIC group being older on average than the control group. The T0 SMDs for baseline outcome variables are reported in Table 3. Exploratory age-adjusted sensitivity analyses showed a generally consistent pattern of Group × Time effects with the primary unadjusted analyses, suggesting that the main findings were unlikely to be solely attributable to baseline age differences.

All 15 participants in the MAIC group attended all scheduled MAIC sessions, corresponding to a session attendance rate of 100%; thus, session attendance was uniform across participants. Based on weekly self-reported adherence checklists, participants reported an average daily home-practice duration of 14.80 ± 2.30 min across the 8-week intervention.

#### 3.1.1. Psychological Stress

A 2 × 3 mixed-design ANOVA was conducted to examine the effects of MAIC training on athlete-specific psychological stress. For APSQ, the analysis revealed a significant group x time interaction effect [*F*(2, 56) = 18.71, *p* < 0.001, partial *η*^2^ = 0.40], and a significant time main effect [*F*(2, 56) = 14.44, *p* < 0.001, partial *η*^2^ = 0.34], whereas the group main effect was not significant [*F*(1, 28) = 0.37, *p* = 0.55, partial *η*^2^ = 0.01]. These findings indicate that changes in psychological stress over time differed significantly between the MAIC and control groups. Descriptive statistics (Table 3) showed that APSQ scores in the MAIC group decreased from 19.87 ± 2.80 at T0 to 16.40 ± 3.14 at T1 and remained lower than baseline at T2 (17.53 ± 2.39), whereas APSQ scores in the control group showed little change across time (18.60 ± 3.07 at T0, 19.00 ± 3.72 at T1, and 18.13 ± 3.25 at T2). Bonferroni-adjusted post hoc comparisons (Table 4) indicated that, in the MAIC group, APSQ scores were significantly lower at T1 than at T0 (*p* < 0.001) and remained significantly lower at T2 than at T0 (*p* = 0.002), whereas the T1–T2 difference was not significant (*p* = 0.09). In contrast, no significant within-group changes were observed in the control group (all *p* > 0.05). Between-group comparisons (Table 5) further showed a significant difference at T1 (*t* = −2.07, *p* = 0.04), but not at T0 (*t* = 1.18, *p* = 0.25) or T2 (*t* = −0.58, *p* = 0.57). Together, these findings suggest that MAIC training reduced athlete-specific psychological stress at post-intervention, with MAIC group scores remaining below baseline at follow-up.

For salivary cortisol, the mixed-design ANOVA revealed a significant group x time interaction effect [*F*(1.34, 37.54) = 4.37, *p* = 0.03, partial *η*^2^ = 0.14], and a significant group main effect [*F*(1, 28) = 9.88, *p* = 0.004, partial *η*^2^= 0.26], whereas the time main effect was not significant [*F*(1.34, 37.54) = 2.25, *p* = 0.14, partial *η*^2^ = 0.07]. Descriptive statistics (Table 3) showed that salivary cortisol levels (nmol/L) in the MAIC group decreased from 34.92 ± 4.82 at T0 to 23.27 ± 4.77 at T1 and remained lower than baseline at T2 (25.60 ± 4.36), whereas the control group showed little change over time (32.98 ± 5.07 at T0, 34.82 ± 6.60 at T1, and 34.66 ± 5.43 at T2). Bonferroni-adjusted post hoc comparisons (Table 4) showed that, in the MAIC group, cortisol levels were significantly lower at T1 than at T0 (*p* < 0.001) and remained significantly lower at T2 than at T0 (*p* = 0.01), whereas the T1–T2 difference was not significant (*p* > 0.05). No significant within-group changes were observed in the control group (all *p* > 0.05). Between-group comparisons (Table 5) indicated no significant difference at T0 (*t* = 1.43, *p* = 0.17), a significant difference at T1 (*t* = −5.49, *p* < 0.001), and a significant difference at T2 (*t* = −5.04, *p* < 0.001). Overall, the salivary cortisol findings were broadly consistent with the APSQ results at post-intervention, while showing a different follow-up pattern: the between-group difference in cortisol remained statistically evident at T2, whereas the corresponding APSQ difference was no longer significant. However, given the small sample size, the exploratory nature of this physiological stress-related measure, and the variability of single awakening cortisol values, these findings should be interpreted cautiously as supportive evidence rather than as definitive evidence of sustained physiological adaptation.

#### 3.1.2. Sleep Quality

A 2 × 3 mixed-design ANOVA was conducted to examine the effects of MAIC training on sleep quality. For PSQI, the analysis revealed a significant Group × Time interaction effect [*F*(2, 56) = 30.59, *p* < 0.001, partial *η*^2^ = 0.52], a significant Group main effect [*F*(1, 28) = 4.22, *p* = 0.049, partial *η*^2^ = 0.13], and a significant Time main effect [*F*(2, 56) = 11.66, *p* < 0.001, partial *η*^2^ = 0.29]. These findings indicate that changes in sleep quality over time differed significantly between the MAIC and control groups. Descriptive statistics (Table 3) showed that PSQI scores in the MAIC group decreased from 4.93 ± 2.63 at T0 to 2.00 ± 1.81 at T1 and remained lower than baseline at T2 (3.87 ± 2.13), whereas PSQI scores in the control group increased from 4.73 ± 2.52 at T0 to 5.60 ± 2.92 at T1 and 5.73 ± 2.60 at T2. Bonferroni-adjusted post hoc comparisons (Table 4) indicated that, in the MAIC group, PSQI scores were significantly lower at T1 than at T0 (*p* < 0.001), whereas the T0–T2 difference was not significant (*p* = 0.10). The T1–T2 comparison was significant (*p* < 0.001), indicating some rebound after the intervention, although scores remained descriptively lower than baseline. In contrast, in the control group, PSQI scores were significantly higher at T1 than at T0 (*p* = 0.04) and at T2 than at T0 (*p* = 0.03), whereas no significant difference was observed between T1 and T2 (*p* > 0.05). Between-group comparisons (Table 5) further showed no significant difference at T0 (*t* = 0.21, *p* = 0.83), but significant differences at T1 (*t* = −4.05, *p* < 0.001), and T2 (*t* = −2.15, *p* = 0.04). Taken together, these findings suggest that MAIC training improved PSQI-assessed subjective sleep quality at post-intervention, with between-group differences still evident at follow-up.

#### 3.1.3. Mindfulness

A 2 × 3 mixed-design ANOVA was conducted to examine the effects of MAIC training on mindfulness. For AMQ, the analysis revealed a significant Group × Time interaction effect [*F*(2, 56) = 19.57, *p* < 0.001, partial *η*^2^ = 0.41], and a significant Time main effect [*F*(2, 56) = 4.21, *p* = 0.02, partial *η*^2^ = 0.13], whereas the Group main effect was not significant [*F*(1, 28) = 2.82, *p* = 0.11, partial *η*^2^ = 0.09]. These findings indicate that changes in mindfulness over time differed significantly between the MAIC and control groups. Descriptive statistics (Table 3) showed that AMQ scores in the MAIC group increased from 62.27 ± 5.96 at T0 to 65.40 ± 5.88 at T1 and remained higher than baseline at T2 (63.60 ± 5.95), whereas AMQ scores in the control group showed little change across time (61.07 ± 5.86 at T0, 59.67 ± 6.59 at T1, and 59.53 ± 6.30 at T2). Bonferroni-adjusted post hoc comparisons (Table 4) indicated that, in the MAIC group, AMQ scores were significantly higher at T1 than at T0 (*p* < 0.001) and remained significantly higher at T2 than at T0 (*p* = 0.001), whereas the T1–T2 difference was also significant (*p* = 0.005), suggesting some decline after the intervention but maintenance above baseline levels. In contrast, no significant within-group changes were observed in the control group (all *p* > 0.05). Between-group comparisons (Table 5) further showed no significant difference at T0 (*t* = 0.56, *p* = 0.58), a significant difference at T1 (*t* = 2.51, *p* = 0.02), and a non-significant difference at T2 (*t* = 1.82, *p* = 0.08). Taken together, these findings suggest that MAIC training increased mindfulness at post-intervention, with MAIC group scores remaining above baseline at follow-up. However, the between-group difference was not statistically significant at T2, and the effect should therefore be interpreted cautiously.

### 3.2. Qualitative Results

Fifteen MAIC-arm athletes completed post-intervention interviews. Analysis generated five higher-order themes with indicative subthemes. Frequency is conveyed qualitatively (e.g., “many”, “some”). Illustrative quotations are presented after each subtheme, translated from Mandarin and cross-checked by a bilingual researcher; participant IDs are anonymized (P01–P15).

**Theme** **1.** *Embedding MAIC under high training load is feasible and acceptable.*

***Fit and Burden:*** Many athletes judged the weekly session plus micro-practices as manageable within dense microcycles; time cost was low when practices were tied to existing cues (e.g., lane turns, post-set recovery).

“The between-set reset just fit into my rest, it didn’t steal training time.”

(P05)

***Sport/Culture Fit:*** Swimming-specific examples (early mornings, two-a-days) felt relatable and credible.

“When examples used our early-morning sets, it clicked, this felt made for us.”

(P12)

***Least Useful Elements:*** A few athletes initially found the insight content abstract without facilitator scaffolding.

“The ‘insight’ part was vague at first, once I was instructed to link it to my whole sport career, it made sense.”

(P03)

**Theme** **2.** *In-training attentional resets and acceptance reduce cognitive reactivity.*

***Present-Moment Attention Under Load:*** Athletes described returning attention to task cues (stroke, breathing) when pace dropped or discomfort rose.

“When my legs burned, I named it and came back to stroke count, pace settled.”

(P07)

***Acceptance:*** Labeling sensations/thoughts (“burn”, “off-pace”) without struggle helped avoid self-criticism spirals and set-to-set rumination.

“Instead of arguing with ‘off-pace’, I let it pass and focused on the next 50.”

(P08)

***Perceived Utility:*** Between-set “reset” and brief breath-anchoring were most frequently cited as most helpful techniques.

“Two breaths, reset, next rep, that tiny routine changed the session.”

(P02)

**Theme** **3.** *Post-session wind-down and pre-sleep routines lower arousal and aid sleep.*

***Down-Regulation After Evening Training:*** Body scan or paced breathing during cooldown/commute helped athletes “switch off”.

“Five minutes of breathing on the bus and my head stopped replaying the last set”.

(P09)

***Pre-Sleep Arousal:*** Many reported fewer intrusive replay thoughts and easier sleep onset on nights with a short routine; some adopted earlier screen curfews.

“If I did the short routine, I fell asleep faster and didn’t loop the splits”.

(P10)

***Limits:*** A minority noted limited change on nights preceding very early training due to constrained sleep opportunity.

“Before a 5 a.m. start, there’s just not enough sleep time, even with the routine.”

(P01)

**Theme** **4.** *Barriers and enablers to sustained practice.*

***Barriers:*** Post-double-session fatigue, noisy environments, travel/competition weeks, and forgetting cues impeded practice.

“After doubles I’m wiped and sometimes forget the practice altogether.”

(P06)

***Enablers:*** Coach reminders, peer norms, cue cards/app audio, and anchoring to fixed routines (e.g., lights-out) supported adherence.

“When the cue card’s on my locker and the coach reminds us, I always do the reset.”

(P13)

***Safety:*** No adverse events were reported; occasional transient emotional reactions early on settled with guidance.

“I felt a wave of emotion in week one, but with guidance it passed quickly.”

(P11)

**Theme** **5.** *Mechanisms and transfer beyond the pool.*

***Proposed Mechanisms:*** Athletes attributed benefits to improved attentional control, acceptance of discomfort, cognitive de-escalation, and values-aligned choices (e.g., prioritizing recovery behaviors).

“MAIC stopped one bad rep from snowballing, I could downshift the thoughts.”

(P04)

***Transfer:*** Skills were applied to school/exams and pre-race nerves; several used brief breathing resets before starts.

“I used the same breathing before an exam, and on the blocks before the start.”

(P14)

***Scalability Suggestions:*** Requests included shorter sessions in taper weeks, periodic boosters, and more stroke-specific examples.

“A short booster during taper and more stroke-specific cues would help.”

(P15)

Across interviews, athletes portrayed MAIC as a low-burden, context-attuned strategy that can be embedded into high-load swimming. Perceived effects clustered around reduced cognitive reactivity during sets and lower pre-sleep arousal, alongside practical barriers linked to fatigue and scheduling. These themes provide a coherent experiential account of how MAIC may contribute to stress reduction and sleep improvements under unchanged physical-activity load, and they inform refinements (e.g., booster dosing, cueing, and taper-phase adaptations) for future implementation.

### 3.3. Mixed-Methods Joint Display

Table 6 presents the joint display used to integrate the quantitative and qualitative findings. The display summarizes how athletes’ qualitative accounts helped explain, extend, or qualify the quantitative results, while also highlighting discordant, limiting, or qualifying considerations.

## 4. Discussion

The present randomized controlled mixed-methods trial examined whether an 8-week MAIC program, embedded within routine high-load swim training, could reduce athlete-specific psychological stress, improve sleep quality, and enhance mindfulness in elite swimmers without altering the underlying training prescription. Overall, the findings were broadly consistent with the study hypotheses. Compared with the control group, swimmers in the MAIC group showed lower psychological strain, better sleep quality, and higher mindfulness at post-intervention. Qualitative accounts further indicated that these benefits were experienced through low-burden, context-specific practices that athletes could incorporate into everyday training and pre-sleep routines. Taken together, these findings suggest that MAIC may represent a feasible and practically relevant mental health support strategy in elite sport contexts characterized by already high physical activity, but persistent psychological and recovery-related demands.

### 4.1. Effects of MAIC on Psychological Stress Under High Training Load

A central finding was that psychological stress decreased in the MAIC group, whereas the control group showed no comparable improvement. This pattern was reflected primarily in self-reported athlete-specific strain (APSQ) and was broadly complemented by salivary cortisol, providing supportive evidence across subjective and physiological stress-related indicators. However, because cortisol was assessed using a single awakening sample at each assessment wave, these values are subject to day-to-day variability and should not be interpreted as robust markers of chronic HPA-axis adaptation. Thus, the salivary cortisol findings are best viewed as preliminary physiological stress-related evidence that supports, rather than definitively confirms, the self-reported stress results. At the same time, the follow-up pattern was somewhat differentiated across indicators: although the between-group difference in APSQ was no longer significant at T2, salivary cortisol remained significantly lower in the MAIC group than in the control group. This suggests that the intervention’s stress-reduction effect was strongest during or immediately after the active training phase, while the maintenance trajectory may have differed for self-reported versus physiological indicators.

This pattern is broadly consistent with the previous literature indicating that mindfulness-based programs can reduce stress-related symptoms and improve psychological functioning in athletic populations (Myall et al., 2023; Noetel et al., 2019; Xie et al., 2025). More specifically, a systematic review and meta-analysis focusing on elite athletes concluded that psychological interventions produced beneficial effects on mental health outcomes overall, while also emphasizing substantial methodological heterogeneity and the need for more ecologically valid trials embedded in real performance settings (W. Wang et al., 2025). A broader meta-analysis of athlete-focused mindfulness-based interventions likewise found that such programs can improve mindfulness and reduce maladaptive affective responses, particularly competitive anxiety and related stress responses (X. Wang et al., 2024). The present findings extend this literature by demonstrating comparable benefits in elite swimmers training under sustained high-load conditions, where stress arises not only from general life demands but also from dense training schedules, early-morning sessions, accumulated fatigue, and performance expectations.

The qualitative findings also offer a plausible explanation for why MAIC may have reduced stress in this context. Athletes repeatedly described using between-set attentional resets, breath anchoring, and acceptance of discomfort to prevent a difficult repetition or a negative thought from escalating into broader self-criticism and cognitive overload. This interpretation is consistent with mindfulness-based sport theory, which proposes that present-moment attention and acceptance may reduce cognitive–emotional reactivity by helping athletes disengage from over-identification with evaluative thoughts and reorient attention toward task-relevant cues (F. L. Gardner & Moore, 2004; Noetel et al., 2019; Rogowska & Tataruch, 2024). In high-load training environments, this may be especially important because sustained fatigue, discomfort, and performance pressure increase psychological load and can make athletes more vulnerable to maladaptive cognitive responses if recovery resources are insufficient (Mellalieu et al., 2021; Sun et al., 2024; Ma et al., 2025). Thus, the qualitative data not only illustrate acceptability but also provide process-level support for the quantitative finding that MAIC was associated with reduced psychological stress during the intervention period.

### 4.2. Effects of MAIC on Sleep Quality Under High Training Load

A second key finding was that PSQI-assessed subjective sleep quality improved in the MAIC group, whereas subjective sleep quality worsened in the control group across the same period. This result is especially relevant in swimmers, because sleep problems in this population are often embedded in the structure of training itself rather than arising incidentally. Early-morning sessions, two-a-day schedules, and heavy training phases can reduce sleep opportunity and disrupt recovery routines (Ashby et al., 2024; Sargent et al., 2014a, 2014b). More broadly, the consensus and review literature have consistently identified athletes as a population vulnerable to insufficient or poor-quality sleep under demanding training and competition conditions (Gupta et al., 2017; Walsh et al., 2021; Halson, 2014). However, because sleep was assessed using the PSQI only, the present findings should be interpreted as changes in perceived sleep quality rather than as evidence of objective improvements in sleep duration, sleep efficiency, or sleep physiology. Against this background, the present findings suggest that MAIC may help athletes manage psychological processes that contribute to perceived sleep disturbance during high-load training phases.

The pattern of results is notable. Whereas PSQI scores in the control group worsened over time, PSQI scores in the MAIC group improved at post-intervention and remained better than the control group at follow-up. This suggests that MAIC may have helped athletes manage post-session and pre-sleep cognitive activation that contributed to poorer perceived sleep quality, rather than demonstrating objective protection against sleep disruption. The qualitative findings align closely with this interpretation. Athletes described body scan and paced breathing as helping them “switch off” after training, reduce intrusive replay thoughts, and fall asleep more easily. This interpretation is consistent with previous work suggesting that mindfulness may improve sleep partly by reducing pre-sleep cognitive and somatic arousal, both of which are closely tied to sleep onset difficulty and perceived sleep quality (Li et al., 2018; Johles et al., 2023; Ong et al., 2012).

This reading is also consistent with the emerging athlete-specific literature. In university athletes, a brief mindfulness induction after evening training reduced pre-sleep arousal and improved subjective sleep quality (Li et al., 2018). Similarly, an 8-week mindfulness-based stress reduction program in female collegiate rowers improved sleep quality alongside broader gains in well-being (Jones et al., 2020). The present study extends this line of evidence into elite swimming, a context in which early starts, accumulated fatigue, and dense training microcycles may make sleep regulation especially challenging.

At the same time, the follow-up pattern warrants a careful interpretation. In the MAIC group, PSQI scores at T2 remained descriptively below baseline but were no longer significantly different from baseline, whereas the between-group difference remained significant because sleep quality in the control group had continued to deteriorate. This suggests that the intervention effect may not have reflected simple linear maintenance of the immediate post-intervention gain. Rather, MAIC may have reduced the likelihood that sleep quality would worsen under ongoing training demands, even if the strongest immediate gains weakened after the structured program ended. From an applied perspective, this pattern points to the potential value of brief booster sessions or continued low-dose practice to help sustain perceived sleep-quality gains over time.

### 4.3. Effects of MAIC on Mindfulness and Its Maintenance

As expected, mindfulness increased significantly in the MAIC group at post-intervention and remained above baseline at follow-up, although some decline from T1 to T2 was evident. This pattern is theoretically coherent, as mindfulness was one of the intended proximal targets of the intervention. It is also broadly consistent with previous sport-focused research suggesting that mindfulness-based interventions can enhance athletes’ mindfulness-related capacities during active intervention phases (Bühlmayer et al., 2017; Y. Wang et al., 2023).

The partial decline at follow-up should not necessarily be interpreted as failure of the intervention. Rather, it may indicate that the maintenance of mindfulness-related gains depends on the extent to which practice continues once structured sessions are withdrawn. Broader mindfulness-intervention research suggests that follow-up benefits are often shaped by ongoing home practice, continued contextual support, and the degree to which mindfulness skills become routinized in everyday life (Parsons et al., 2017; Ribeiro et al., 2018). A similar implication emerges from follow-up work showing that mindfulness-related gains can remain meaningful over time, but are more likely to be sustained when participants retain opportunities for continued engagement and reinforcement (Galante et al., 2021; Lymeus et al., 2022; Miles et al., 2023; Thompson et al., 2011).

The qualitative findings deepen this interpretation. Athletes identified brief breath-based resets and cue-linked practices as the most useful components, but they also requested booster sessions, taper-phase reinforcement, and more stroke-specific examples. This suggests that maintenance of mindfulness under elite training conditions may depend not only on initial acquisition, but also on whether the practices remain practically embedded in the rhythms of daily sport. In high-performance swimming, where athletes return quickly to demanding schedules and recovery constraints after formal intervention ends, some attenuation may therefore be expected if no maintenance structure is provided. From this perspective, the non-significant between-group difference in AMQ at T2, despite the MAIC group remaining above its own baseline, may reflect not the disappearance of benefit, but the challenge of sustaining mindfulness gains in a high-load environment without continued reinforcement. This interpretation is consistent with sport mindfulness theory, which emphasizes that attentional and acceptance-based skills are most likely to endure when they are repeatedly enacted in context rather than treated as one-off classroom content (Bühlmayer et al., 2017; F. L. Gardner & Moore, 2004, 2017; Gutman et al., 2025).

### 4.4. Integrated Interpretation of the Mixed-Methods Findings

As summarized in the mixed-methods joint display (Table 6), the mixed-methods findings provide a more coherent understanding of the role of MAIC than either strand could offer alone. Quantitatively, the intervention was associated with reduced psychological stress, improved subjective sleep quality, and increased mindfulness during the active intervention period. Qualitatively, athletes described these changes in terms of concrete, training-embedded processes, including between-set attentional resets, greater acceptance of discomfort, and brief post-session or pre-sleep down-regulation practices. The qualitative strand therefore helped explain and qualify the quantitative pattern by identifying both the practical self-regulatory processes through which MAIC was enacted and the contextual limits that shaped its use. Considered together, these findings suggest that MAIC was experienced not as an additional burden alongside training, but as a set of low-demand regulatory skills that could be incorporated into the existing structure of elite swimming. This interpretation is also consistent with sport mindfulness theory, which suggests that mindfulness-based training may support performance and well-being by strengthening present-moment attention, reducing entanglement with evaluative thoughts, and promoting acceptance-based self-regulation under pressure (F. L. Gardner & Moore, 2004; Su et al., 2019).

This integrated pattern is especially relevant in the present context of high physical activity and sustained training load. In such settings, the challenge is unlikely to be insufficient physical activity itself, but rather the psychological strain and recovery disruption that can accompany intensive training. From this perspective, MAIC may be understood as complementing, rather than competing with, the training process by helping athletes regulate cognitive–emotional reactivity during training and reduce arousal during the transition to sleep. At the same time, the joint display also highlights limiting or less favorable cases that temper this interpretation: some athletes found the insight-related content initially abstract, post-double-session fatigue and competition-related disruption sometimes impeded practice, and constrained sleep opportunity before very early training limited perceived sleep benefits for some athletes. Although the present design does not permit strong causal claims about mechanism, the convergence between the quantitative and qualitative findings offers an athlete-grounded explanation of how mindfulness-based support may function under ecologically valid high-performance conditions. Thus, the integrated findings should be understood as explanatory rather than merely confirmatory: they indicate how MAIC may have supported stress regulation, subjective sleep quality, and mindfulness, while also identifying implementation boundaries for future refinement. This kind of explanatory integration is consistent with the broader rationale of mixed-methods research, in which qualitative data help clarify how and why quantitative changes may occur in practice (Fetters et al., 2013; Creswell & Plano Clark, 2018).

The present findings also help clarify why MAIC may be especially well matched to elite swimming. Swimming training is highly repetitive, physically demanding, and rhythmically structured around breath, stroke, pace, interval timing, and rest periods. These features may make swimmers vulnerable to rumination and self-evaluative reactivity when pace drops or fatigue accumulates, but they also provide stable cues for brief mindfulness–acceptance practices. In this study, athletes’ accounts suggested that MAIC was most useful when skills were attached to existing swimming routines, such as between-set resets, breathing during cooldown, and pre-sleep wind-down after evening training. The Insight component may further support swimmers by helping them reinterpret transient off-pace performances, fatigue, or discomfort in relation to broader athletic values, rather than treating each difficult set as evidence of failure. This sport-specific fit may explain why MAIC was perceived as low burden and practically usable under high-load conditions.

### 4.5. Practical Implications

The present findings have several practical implications for high-performance sport settings. First, the results support MAIC as a low-burden, ecologically deployable intervention that can be integrated into elite swimming programs without altering the underlying training prescription. This is important for applied implementation, because interventions that minimize disruption to established training routines are more likely to be feasible and acceptable within elite sport environments, where time, recovery, and coaching priorities are tightly constrained (Bender & Lambing, 2024; Bernier et al., 2025). Second, the qualitative findings suggest that the most scalable elements of MAIC may not be the longest formal sessions, but rather the briefest and most context-linked practices, such as between-set resets, cooldown breathing, and short pre-sleep wind-down routines. These kinds of embedded strategies appear especially relevant in sports such as swimming, where early-morning training, dense schedules, and accumulated fatigue can make additional standalone activities difficult to sustain (Sargent et al., 2014a, 2014b; Walsh et al., 2021). Third, the partial attenuation of effects at follow-up suggests that future implementation may benefit from booster sessions or light-touch maintenance modules during taper periods, travel blocks, or other phases associated with elevated stress and disrupted sleep. This recommendation is consistent with athlete sleep guidance, emphasizing that practical, targeted, and context-sensitive strategies are more likely to support recovery in real-world performance environments (Bender & Lambing, 2024; Halson, 2019).

### 4.6. Limitations and Future Directions

Several limitations should be acknowledged. First, the sample was relatively small and drawn from a single provincial high-performance swimming program, which may limit the generalizability and statistical precision of the findings. Although the study was designed to preserve ecological validity within an intact elite squad, the limited sample size means that the estimated effects should be interpreted cautiously. In particular, the partial *η*^2^ values observed for several Group × Time interactions may be unstable and potentially inflated, as effect-size estimates derived from small samples can overestimate the magnitude of the true population effect. In addition, non-significant baseline *p*-values should not be interpreted as evidence of full equivalence in a small randomized sample. The MAIC group was older on average than the control group, and age may influence stress perception, emotional regulation, sleep behavior, and responsiveness to mindfulness-based training. Although exploratory age-adjusted sensitivity analyses showed a generally consistent pattern of findings, residual confounding related to age or other demographic characteristics cannot be fully ruled out. The present findings should therefore be viewed as preliminary evidence of the feasibility and potential benefits of MAIC, rather than as definitive estimates of intervention efficacy. Larger multisite trials with conventional power levels, stratified randomization or minimization procedures, and prespecified covariate-adjusted analyses are needed to obtain more stable effect estimates and to determine whether the observed effects replicate across broader athlete populations and training contexts. Second, although a three-month follow-up was included, this period remained relatively short to evaluate the durability of intervention effects across different training phases and competitive cycles. Third, the cortisol and sleep measures should be interpreted with caution. Salivary cortisol was based on a single awakening sample at each assessment wave rather than a multi-sample cortisol awakening response (CAR) protocol; therefore, it should be interpreted as single-sample awakening cortisol rather than as a dynamic CAR index. This approach may be more sensitive to day-to-day contextual variability and limits inference about sustained HPA-axis adaptation. Sleep outcomes were assessed using the PSQI, a subjective self-report measure, and no objective sleep monitoring was included. Therefore, the observed sleep-related findings should be interpreted as changes in perceived sleep quality rather than as evidence of objective improvements in sleep duration, sleep efficiency, sleep architecture, or sleep physiology. Future studies would benefit from combining validated questionnaires with multi-sample CAR protocols, objective indicators, or repeated daily sleep assessments, such as actigraphy, wearable-derived sleep metrics, or sleep diary protocols. Fourth, placebo expectancy and self-report bias cannot be fully ruled out. Because the MAIC group received a structured psychological intervention with regular instructor contact, whereas the control group continued usual practice, some self-reported improvements may have been influenced by expectancy, perceived support, demand characteristics, or social desirability. Future trials should consider attention-matched or active control conditions, direct assessment of treatment expectancy, and more objective or repeated daily measures where feasible. Fifth, although the qualitative component provided valuable insight into perceived processes, implementation, and limiting cases, interviews were conducted only with athletes in the MAIC group and therefore did not capture control-group perspectives or allow a balanced between-group qualitative comparison of experiences during the same high-load training period. Accordingly, the qualitative findings should be interpreted primarily as explaining and qualifying intervention-group experiences, rather than as providing a balanced qualitative comparison between MAIC and usual practice. Future mixed-methods trials should include qualitative sampling from both intervention and control participants to better examine convergent and divergent experiences across groups. Finally, because some effects were attenuated by follow-up, future trials should examine whether booster sessions or maintenance-oriented adaptations can help sustain gains over time. More broadly, future studies should evaluate the transferability and long-term sustainability of MAIC across diverse high-performance sport contexts.

## 5. Conclusions

In conclusion, this randomized controlled mixed-methods trial indicates that an 8-week MAIC program, when embedded within routine high-load swim training, may help reduce athlete-specific psychological stress, improve subjective sleep quality, and enhance mindfulness in elite swimmers. The qualitative findings further suggest that these benefits were experienced through feasible, low-burden practices that helped athletes regulate cognitive reactivity during training and down-regulate arousal before sleep. Taken together, the findings support the feasibility and potential utility of MAIC as a culturally grounded and practically viable adjunct strategy for supporting psychological regulation and subjective sleep quality in elite swimming, without disrupting the training load required for performance preparation. Future research should examine MAIC in larger multisite samples, over longer follow-up periods, and with maintenance boosters and more objective sleep assessment.

## Figures and Tables

**Table 1:** Eight-week MAIC course outline (embedded within routine high-load swim training).

Week	Theme	Key In-Session Elements (≈90 min Group Session)	Home Practice (≈15 min Daily)
1	Orientation to MAIC	Program overview; rationale for use during high-load phases; breath anchoring; brief seated practice; personal goal-setting	Intro audio (breath anchor or short body scan)
2	Foundations of mindfulness	Focused vs. open monitoring; body scan; mindful movement; basic “present-moment” cues for training days	Body scan or breath anchor (alternate days)
3	Attention and awareness under load	Detecting attentional drift during demanding sets; cueing present-moment focus; short open-monitoring practice	Open monitoring (5–10 min) + 3–5 min breath close
4	Acceptance and cognitive defusion	Experiential avoidance vs. acceptance; defusion scripts for “hard-set” thoughts; brief sit	Acceptance/defusion audio (e.g., leaves-on-a-stream)
5	Values clarification	Clarify athletic/personal values; link values to daily training choices; plan value-aligned micro-actions	5–15 min values reflection + breath anchor
6	Insight	Insight (loosening rigid cognitive sets and fostering flexible, values-aligned appraisal); decentering from rumination/perfectionism	Decentering practice (5–8 min) + brief journaling
7	Committed action and sleep wind-down	Committed action under fatigue; model of pre-sleep arousal; build a simple wind-down routine	Pre-sleep wind-down audio (breath to brief body scan)
8	Consolidation and relapse-prevention	Review of core skills; individualized “if–then” plans for meets/travel; maintenance schedule	Choice of any core audio; 5 min values check

Note. MAIC, Mindfulness–Acceptance–Insight–Commitment.

**Table 2:** Semi-structured interview guide (domains and exemplar prompts).

Domain	Sample Prompts (Examples)
Baseline context (pre-MAIC)	Main stressors around training/competition? Typical sleep during heavy phases or early-morning/double sessions?
Acceptability and fit	How well did MAIC sessions/homework fit your schedule? Was the content clear and credible? Any cultural/sport-specific fit issues?
In-training application	Which MAIC skills were used during workouts? In which sets or moments?
Post-training and pre-sleep routines	What, if anything, was practiced after sessions or before bedtime to wind down or handle rumination/pre-sleep arousal?
Perceived effects and technique utility	Noticed changes in stress, focus, recovery, or sleep? Which technique felt most/least helpful, and in what situations?
Barriers and enablers	What made practice hard or easy (time pressure, fatigue, environment, staff/teammate support, materials/apps)?
Adherence and transfer	How often did you practice outside sessions? Did skills transfer to life or competition? What cues helped you keep it up?
Perceived mechanisms	In your view, why did MAIC help (or not) with stress or sleep (awareness, attention, acceptance, cognitive de-escalation, values-aligned action)?
Safety and unintended effects	Any adverse or uncomfortable experiences? How were they handled?
Suggestions and scalability	What would you change about MAIC content, timing, or delivery? Would you recommend MAIC to a teammate, why/why not?

Note. The guide was used flexibly; additional topics raised by participants were explored as appropriate.

**Table 3:** Descriptive statistics of the aimed variables at three data points.

Variables	T0			T1		T2	
	*M*	*SD*	*SMD*	*M*	*SD*	*M*	*SD*
**Psychological Strain**							
MAIC	19.87	2.80	0.43	16.40	3.14	17.53	2.39
CG	18.60	3.07		19.00	3.72	18.13	3.25
**Salivary Cortisol** (nmol/L)							
MAIC	34.92	4.82	0.39	23.27	4.77	25.60	4.36
CG	32.98	5.07		34.82	6.60	34.66	5.43
**Sleep Quality**							
MAIC	4.93	2.63	0.08	2.00	1.81	3.87	2.13
CG	4.73	2.52		5.60	2.92	5.73	2.60
**Mindfulness**							
MAIC	62.27	5.96	0.20	65.40	5.88	63.60	5.95
CG	61.07	5.86		59.67	6.59	59.53	6.30

Note. *M* = Mean; *SD* = standard deviation; *SMD* = standardized mean difference; MAIC = MAIC group; CG = control group; SMDs were calculated as MAIC minus CG for T0 values, where positive values indicate higher MAIC scores; sleep quality was assessed using the PSQI and refers to self-reported sleep quality.

**Table 4:** Summaries of Bonferroni-adjusted post hoc comparisons.

Variables	T0 vs. T1		T0 vs. T2		T1 vs. T2	
	*M diff*	*p*	*M diff*	*p*	*M diff*	*p*
**MAIC**						
Psychological Strain	3.47	<0.001	2.33	0.002	−1.13	0.09
Salivary Cortisol (nmol/L)	11.65	<0.001	9.33	0.01	−2.33	0.22
Sleep Quality	2.93	<0.001	1.06	0.10	−1.87	<0.001
Mindfulness	−3.13	<0.001	−1.33	0.001	1.80	0.005
**CG**						
Psychological Strain	−0.4	0.73	0.47	0.33	0.87	0.27
Salivary Cortisol (nmol/L)	−1.84	0.31	−1.68	0.43	0.16	0.92
Sleep Quality	−0.87	0.04	−1.00	0.03	−0.13	0.94
Mindfulness	1.40	0.12	1.53	0.14	0.14	0.93

Note. *M diff* = mean difference; *p* = *p* value; MAIC = MAIC group; CG = control group; *M diff* was calculated as the earlier time point minus the later time point for each comparison; positive values indicate higher scores at the earlier time point. Sleep quality was assessed using the PSQI and refers to self-reported sleep quality.

**Table 5:** Summaries of between-group comparisons.

Variables	T0			T1				T2			
	*t*	*p*	*df*	*t*	*p*	*df*	95% *CI*	*t*	*p*	*df*	95% *CI*
Psychological Strain	1.18	0.25	28	−2.07	0.04	28	[−5.17, −0.03]	−0.58	0.57	28	[−2.73, 1.53]
Salivary Cortisol (nmol/L)	1.43	0.17	18.98	−5.49	<0.001	28	[−15.86, −7.24]	−5.04	<0.001	28	[−12.74, −5.38]
Sleep Quality	0.21	0.83	28	−4.05	<0.001	28	[−5.42, −1.78]	−2.15	0.04	28	[−3.65, −0.09]
Mindfulness	0.56	0.58	28	2.51	0.02	28	[1.06, 10.41]	1.82	0.08	28	[−0.52, 8.65]

Note. *t* = *t* value; *p* = *p* value; *df* = degree of freedom; *CI* = confidence interval; sleep quality was assessed using the PSQI and refers to self-reported sleep quality; the 95% CI refers to the between-group mean difference calculated as MAIC minus CG, where negative values indicate lower MAIC scores and positive values indicate higher MAIC scores; sleep quality was assessed using the PSQI and refers to self-reported sleep quality.

**Table 6:** Joint display integrating quantitative and qualitative findings.

Quantitative Finding	Related Qualitative Finding	Integrated Inference	Discordant/Limiting/Qualifying Considerations
Psychological strain decreased in the MAIC group during the active intervention period	Athletes described between-set attentional resets, breath anchoring, and acceptance of discomfort as helping them return attention to task cues and reduce rumination after difficult repetitions.	Qualitative findings help explain the stress reduction as a process of reduced cognitive reactivity and improved in-training self-regulation under high load.	Some athletes reported that fatigue after double training sessions made it difficult to remember or sustain practice.
Salivary cortisol findings broadly complemented the self-reported stress pattern	Athletes described less escalation from negative thoughts and greater ability to downshift cognitive–emotional activation during and after training.	The physiological stress-related findings are consistent with athletes’ accounts of lower perceived activation, but should be interpreted as supportive rather than definitive evidence.	Cortisol was assessed using a single awakening sample at each wave; therefore, physiological interpretation remains preliminary.
Self-reported sleep quality improved in the MAIC group, with between-group differences still evident at follow-up	Athletes described post-session breathing, body scan, and pre-sleep routines as reducing replay thoughts and helping them switch off after training.	Qualitative findings provide a plausible explanation that MAIC supported perceived sleep quality by reducing pre-sleep cognitive activation.	A minority noted limited change before very early training sessions because sleep opportunity was constrained.
Mindfulness increased during the active intervention period.	Athletes reported using present-moment attention, acceptance, and cognitive defusion to avoid over-identifying with off-pace thoughts or discomfort.	Qualitative findings explain mindfulness gains as skills that were enacted in concrete swimming-specific contexts rather than only as abstract psychological concepts.	A few athletes initially found insight-related content abstract until it was linked more explicitly to their sport career and training experience.
Intervention adherence was high.	Athletes described MAIC as low burden when practices were tied to existing training cues, such as rest intervals, cooldown, or lights-out routines.	Feasibility was supported by embedding MAIC into existing training and recovery routines rather than adding a separate heavy intervention burden.	Barriers included fatigue, noisy environments, travel or competition weeks, forgetting cues, and requests for shorter taper-week sessions or booster sessions.

Note. The joint display summarizes how qualitative themes helped explain, extend, or qualify the quantitative findings. Qualitative interviews were conducted only with MAIC-group athletes; therefore, integrated inferences primarily reflect intervention-group experiences.

## Data Availability

The original contributions presented in this study are included in the article. Further inquiries can be directed to the corresponding author.
